# Frequency unlocking-based MEMS bifurcation sensors

**DOI:** 10.1038/s41378-023-00522-2

**Published:** 2023-05-16

**Authors:** Yan Qiao, Zhan Shi, Yutao Xu, Xueyong Wei, Alaaeldin Elhady, Eihab Abdel-Rahman, Ronghua Huan, Wenming Zhang

**Affiliations:** 1 State Key Laboratory of Mechanical System and Vibration, School of Mechanical Engineering, Shanghai Jiao Tong University, Shanghai, China; 2 Department of Mechanics, Key Laboratory of Soft Machines and Smart Devices of Zhejiang Province, Zhejiang University, Hangzhou, China; 3 State Key Laboratory for Manufacturing Systems Engineering, Xi’an Jiaotong University, Xi’an, China; 4 Department of Systems Design Engineering, University of Waterloo, Waterloo, ON Canada

**Keywords:** Engineering, Electrical and electronic engineering

## Abstract

MEMS resonators exhibit rich dynamic behaviors under the internal resonance regime. In this work, we present a novel MEMS bifurcation sensor that exploits frequency unlocking due to a 1:3 internal resonance between two electrostatically coupled micro-resonators. The proposed detection mechanism allows the sensor to operate in binary (digital) and analog modes, depending on whether the sensor merely detects a significant jump event in the peak frequency upon unlocking or measures the shift in the peak frequency after unlocking and uses it in conjunction with a calibration curve to estimate the corresponding change in stimulus. We validate the success of this sensor paradigm by experimentally demonstrating charge detection. High charge resolutions are achieved in binary mode, up to 0.137 fC, and in analog mode, up to 0.01 fC. The proposed binary sensor enables extraordinarily high detection resolutions due to the excellent frequency stability under internal resonance and the high signal-to-noise ratio of the shift in peak frequency. Our findings offer new opportunities for high-performance ultrasensitive sensors.

## Introduction

MEMS-resonant sensors have demonstrated their tremendous potential in both fundamental science and industrial applications. They can be used in weak force, mass, and field sensing, due to significant advantages including ultrasmall mass and high resonant frequencies, that enables ultralow detection limits and superior sensitivities^1,2^. The conventional sensing approach is based on quantitative variations in the linear response that relates a measurable shift in the resonant frequency of the sensors to the bonding of a target stimulus^3^. However, some limits, including the limited signal-to-noise ratio (SNR), the frequency uncertainty, or the effective nonlinearity, inevitably degrade the detection resolution and sensitivity of these sensors that operate in linear regimes^4–6^.

Resonant sensors operating in the nonlinear regime have attracted increasing attention due to their larger output signals increasing the SNR, as well as intriguing nonlinear dynamic features, such as multivalued responses^7^, bifurcations^8–10^, and various types of nonlinear resonances^11–13^. In this regime, various detection principles have been proposed to enhance the performance of resonant sensors, such as tracking the shift in the nonlinear resonant frequency^14^ or exploring the bifurcation phenomena^15–17^. In particular, bifurcation-based sensors have attracted significant interest in recent years. The underlying mechanism of bifurcation sensors is that the target stimulus causes the sensor to cross a bifurcation point, creating a sudden (discontinuous) jump in the response amplitude^16^ or a qualitative change in the phase angle^10^. This paradigm allows the sensor to operate in either binary (detector) or analog modes, depending on the bifurcation detection approaches. The analog mode can be achieved by sweeping the excitation frequency toward the bifurcation to trigger a qualitative change event and then measuring the quantitative change in the bifurcation frequency^18^. On the other hand, a binary sensor operates at a constant excitation frequency just below the bifurcation point while monitoring the qualitative change event of the sensor amplitude as a detector of a single event that stimulus change exceeds a threshold^16^. Unfortunately, because the resonant frequency has a strong dependence on the oscillation amplitude (amplitude-frequency (a-f) effect) in the nonlinear regime^19^, this unavoidably results in the transformation of amplitude fluctuations into frequency fluctuations. Therefore, it will increase the frequency noise of the resonator, and as a consequence, noise-activated stochastic switching between qualitatively different responses (the sensor detection metric) is triggered^20,21^, which represents a false-positive^22^.

Another remarkable nonlinear phenomenon is internal resonance (IR), which arises due to strong modal interactions and intermodal energy transfer^23–25^. It occurs among different vibration modes of a specially designed structure^26^ or between coupled multi-resonator structures (a system)^27^ when the following conditions are satisfied: (i) the natural frequencies of distinct vibrational modes in a single resonator or across coupled resonators are commensurate or nearly commensurate with each other and (ii) a proper type of nonlinearity in accordance with the frequency ratio exists in the resonators. A strong intermodal coupling in IR results in an effective and fast energy transfer between the two modes. As a result, the undriven mode drains mechanical energy from the externally excited mode and vibrates at its own frequency, thereby causing the amplitude of the externally excited mode to drop to a lower level. IR provides an alternative strategy to steer energy in micromechanical resonators and, thus, can be used as negative feedback to stabilize frequency fluctuations^28^. Antonio et al. ^26^ demonstrated that the frequency of the fundamental vibrational mode can be stabilized by coupling it to a higher mode through IR, as the energy exchange between two modes causes the resonance of the higher mode to absorb the amplitude and frequency fluctuations of the first vibrational mode.

Moreover, IR can also be employed for sensor applications, such as introducing an additional sensing channel to detect two different physical quantities simultaneously^29^, sensing the angular rate^30^ or the mass^31,32^, and detecting a single electron^33^. Zhang et al. ^34^ reported that the resonant frequency shift of the fundamental mode could be magnified integer times in the higher modes via IR, leading to an enhanced sensitivity in frequency-shift-based sensors.

Internal resonances in electrostatically or mechanically coupled multi-resonators have unique advantages compared to those of a single resonator. It does not involve different vibrational modes, making the measurement of each element easier and the IR more controllable and flexible for practical applications. On the other hand, the use of coupled multi-resonators also has the benefit of improving common mode rejection capabilities, thereby being less susceptible to environmental noise^35^. Wang et al. ^27^ investigated the IR between two electrostatically coupled micro-resonators with a natural frequency ratio of 1:3 and the resulting phenomena of frequency locking and amplitude saturation, resulting in a sevenfold improvement in frequency stability. Shi et al. ^36^ also studied the 1:3 IR-induced amplitude and frequency locking in two mechanically coupled nonlinear resonators, with nearly an order of magnitude improvement in amplitude stability. Therefore, it is suitable as an amplitude-based sensor.

In this work, we explore the potential for the use of IR in two electrostatically coupled micro-resonators with a natural frequency ratio of nearly 1:3 to design a novel bifurcation sensor. The sensor can function as a binary detector to detect the target stimuli in excess of a given (safe) value by exploiting the transition (jump) in the nonlinear resonance frequency from locking to unlocking. It can also serve as an analog sensor to quantify the stimuli change explicitly by measuring the changes in the peak frequency after unlocking. We present experimental measurements of the IR response of the coupled resonators to interpret the frequency locking and unlocking behaviors. We derive a closed-form expression for the size of the peak frequency shift after unlocking using the multiple-scale method. Then, we experimentally demonstrate an electrometer to validate the proposed detection principle. Finally, we report the sensor’s outcomes and limitations.

## Results and discussion

### Device and characterization

The micro-resonators under test were fabricated through a commercial silicon-on-insulator (SOI)-MUMPs micromachining process, presented in Fig. S1. The two resonators labeled R1 and R3 were made of double-ended tuning forks (DETFs), as shown in Fig. S2, with dimensions of $550 \times 7 \times 25\mu m$ and $292 \times 7 \times 25\mu m$, respectively, for the length, width, and height. The endings of the tuning fork are anchored in gold electrodes to connect with external electrical sources and circuits. The gold electrodes in the mid-span are utilized for electrostatic actuation and detection across a capacitive gap of $3\mu m$. The parallel plates between the two tuning forks are utilized to produce an electrostatic coupling force by exerting different voltages on two plates separately across a capacitive gap of $3\mu m$. A summary of the dimensions and design parameters assigned for the resonators is given in Fig. S3 and Table S1.

**Fig. 1:**
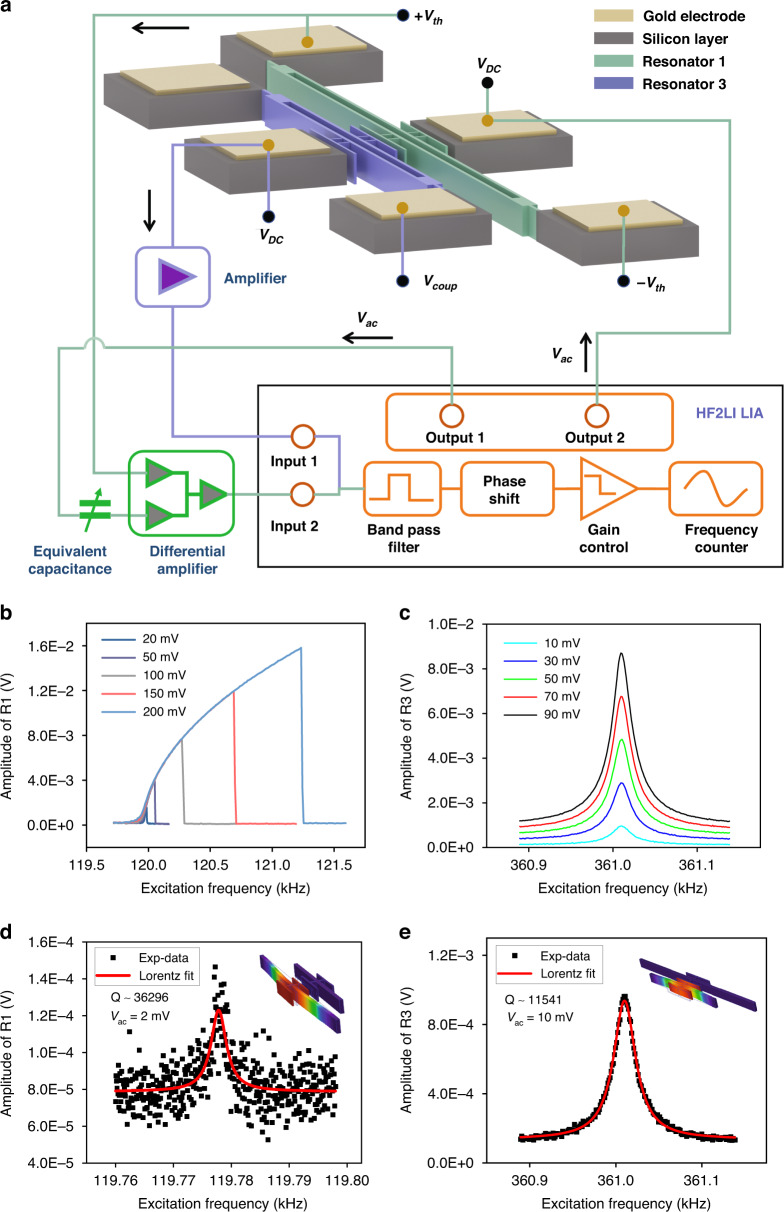
**Characterization of the uncoupled resonators.** **a** A diagram of the measurement circuit in an open-loop configuration. **b**, **c** The frequency-response curves of uncoupled resonators R1 and R3, respectively. **d**, **e** The linear responses of R1 and R3, respectively, where the insets are the corresponding primary mode shapes. The measured quality factors of the two resonators are $Q_1 = 36296$ and $Q_3 = 11541$, respectively

The experimental setup is shown in Fig. S4. The device-under-test (DUT) was placed inside a vacuum chamber under 10^−2^ Pa of pressure at room temperature. A power supply was used to apply the desired DC voltage signal. The Zurich Instruments lock-in amplifier (LIA-HF2LI) was used to apply the desired AC voltage of the drive signal and extract the output signal in combination with an external measurement circuit, as shown in Fig. 1a.

The basic features of the uncoupled two resonators were tested first. Each resonator was electrostatically excited by applying an electric voltage of $V(t) = V_{dc} + V_{ac}\sin (\omega t)$ to the side electrode in the mid-span, where $V_{dc}$ is the DC bias and $V_{ac}$ is the amplitude of the applied AC voltage. The motion of each resonator was detected via piezoresistive transduction. Resonators made of monocrystalline silicon intrinsically have a piezoresistive effect and can serve as piezoresistors. The schematic of the piezoresistive detection is shown in Supplementary S4. The original current through the resonator was generated by applying a DC voltage to one of the anchor pads of the resonator. The vibration amplitudes of the resonators were measured by monitoring the dynamic current. Amplifiers were used to convert the dynamic current to voltage output signals. The parasitic feedthrough signal^37^ intrinsic to all electrically interfaced MEMS resonators can lead to a distortion of the output signal. However, it can be eliminated via an equivalent capacitance generated by adding an adjustable capacitor and a differential operation in the amplifier, as shown in the green block in Fig. 1a.

The natural frequencies of resonators R1 and R3 were evaluated from their linear frequency-response curves and found to be $\omega _1$ = 136 kHz and $\omega _3$ = 361 kHz, respectively. However, to experimentally obtain a 1:3 IR, we need $3\omega _1 < \omega _3$; thus, by driving R1 with an appropriate force, it can be made to resonate at a frequency equal to (1/3) $\omega _3$. Therefore, we use electrothermal actuation by passing an electrical current generated by loading the constant DC voltages $\pm V_{th} = \pm 23.7 V$ on the ends of R1, as shown in Fig. 1a. This heats the resonator through Joule heating and induces an axial stress inside it^38^, tuning (softening) the natural frequency of R1 to 119.78 kHz, Fig. 1d, which is slightly lower than one-third of the natural frequency of R3. The frequency-response curves of R1 (Fig. 1b) and R3 (Fig. 1c) show that R1 exhibits a strong Duffing-like nonlinearity, while R3 is essentially linear within their respective motion domains. The quality factors of R1 and R3 are measured as 36 296 and 11 541, respectively, from their linear frequency response under a relatively small-amplitude excitation, using the half-power bandwidth method, as shown in Fig. 1d, e.

### Frequency locking and unlocking

Next, we investigate the phenomena of frequency locking and unlocking under a 1:3 IR between the two coupled resonators. A DC voltage of $V_C = 8V$ is loaded on one of the anchor pads of resonator R3 to produce a coupling voltage $V_C$ between R1 and R3, where the coupling force is proportional to $V_C^2$. Resonator R1 is electrostatically excited by a biased waveform with $V_{dc} = 20V$ and various AC voltages, while no excitation is applied to resonator R3. The motion of R1 is detected via piezoresistive transduction, while that of R3 is detected capacitively, as shown in Fig. 1a.

**Fig. 2:**
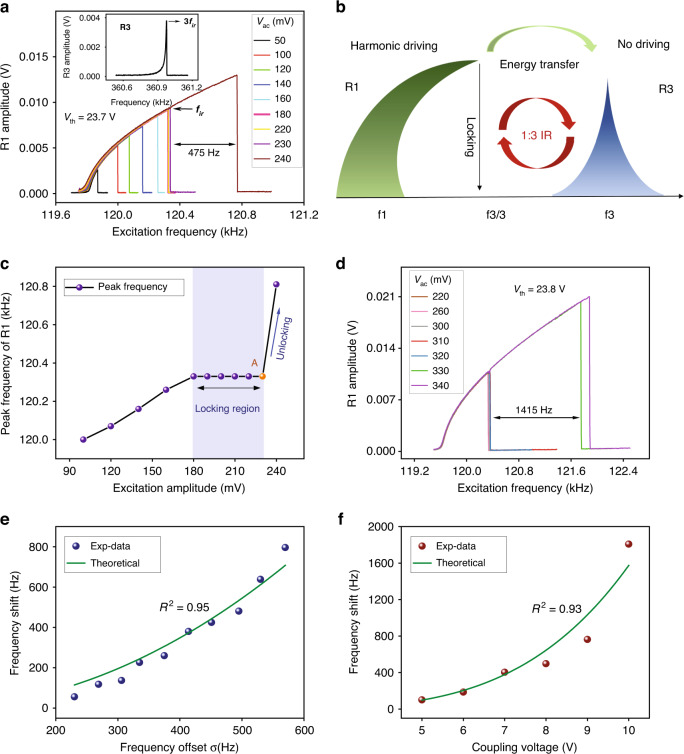
**Frequency locking and unlocking behaviors under a 1:3 IR.** **a** The frequency-response curves of resonator R1 under bias DC voltage $V_{dc} = 20V$, electrothermal voltage $V_{th} = 23.7V$, coupling voltage $V_C = 8V$ and varied excitation amplitudes. Inset: the frequency-response curve of resonator R3 due to IR. **b** A schematic of the 1:3 internal resonance between two coupled resonators. **c** The peak frequency versus excitation amplitude $V_{ac}$ of resonator R1, extracted from **a**. **d** The frequency-response curves of resonator R1 under $V_{th} = 23.8V$. The dependence of the measured peak frequency-shift $\Delta f$ of resonator R1 from locking to unlocking, at an excitation amplitude of 10 mV above the critical value, on the (**e**) initial frequency offset $\sigma$ (blue circles) between the natural frequency and the locked frequency of R1 and (**d**) coupling voltage $V_C$ between two resonators (reddish-brown circles). The theoretical predictions of Eq. (13) are shown as solid green lines

The frequency-response curves in the vicinity of the primary resonance of resonator R1 were obtained by performing a forward frequency sweep under varied excitation amplitudes, as shown in Fig. 2a. The curves bend to the right due to the dominance of the mechanical hardening nonlinearity. As the excitation amplitude $V_{ac}$ varies from 50 mV to 180 mV, the peak amplitude and frequency increase, as expected in a Duffing resonator. When $V_{ac}$ increases from 180 mV to 230 mV, the response falls to the lower branch at the same frequency $f_{ir} = 120.335{\kern 1pt}$ kHz ($f_{ir} = f_3/3$), indicating the activation of IR. At this point, a strong interaction between R1 and R3 is achieved and, as a result, R3 drains more energy from R1, reaching its maximum vibration amplitude, as shown in the inset of Fig. 2a, which locks both the amplitude and frequency of oscillation of R1. Fig. 2b provides a graphical interpretation of this relationship.

When $V_{ac}$ is further increased to 240 mV, the end of the IR condition is reached and the response of R1 unlocks and jumps back to the values expected for the Duffing resonator while R3 almost stops vibrating. Although both the peak frequency and amplitude of R1 undergo a large and discrete surge from locking to unlocking, the former is more remarkable, in which a shift of 475 Hz is observed. The dependence of the peak frequency on the excitation amplitude $V_{ac}$, extracted from Fig. 2a, is shown in Fig. 2c, where a frequency locking region and a bifurcation point A, corresponding to the locked frequency and the critical (unlocking) excitation amplitude, are observed. This suggests the possibility of implementing a bifurcation sensor that exploits the marked jump in the peak frequency across the bifurcation point as a detection mechanism.

In Fig. 2d, we show the effect of the electrothermal voltage loaded on the ends of the resonator R1 on frequency locking and unlocking as $V_{th}$ is changed from $23.7V$ to $23.8V$. In this case, the natural frequency of R1 is reduced, and thus, the critical excitation amplitudes $V_{ac}$ needed to activate and closure the internal resonance are increased. Consequently, the shift in the peak frequency from locking to unlocking is observed to be 1415 Hz at an excitation amplitude of 10 mV above the critical value. We conclude that the peak frequency shift beyond the bifurcation increases with the initial frequency offset $\sigma$ between the natural frequency $f_1$ of R1 and the locked frequency $f_{ir}$($\sigma = f_{ir} - f_1$).

Next, we further examine the impact of the interaction between the initial frequency offset $\sigma$ and coupling voltage $V_C$ on the peak frequency shift $\Delta f$ of R1 from locking to unlocking. First, we hold the coupling voltage fixed at $V_C{{{\mathrm{ = }}}}8V$ while varying the initial frequency offset $\sigma$ of R1 by increasing the electrothermal voltage $V_{th}$ applied to it. The procedure followed to generate Fig. 2d was adopted at different initial frequency offsets $\sigma$. The peak frequency-shift $\Delta f$ at an excitation amplitude of 10 mV above the critical value was recorded and shown as blue circles in Fig. 2e. The frequency-shift value increases as the initial frequency offset increases. Alternatively, we hold the initial frequency offset constant at 500 Hz and vary the coupling voltage between two resonators to examine the peak frequency-shift, Fig. 2f. A gradual increase in the frequency shift with the coupling voltage is observed and shown as reddish-brown circles.

To further interpret those relationships, we derive a closed-form expression for the size of the peak frequency-shift after unlocking (see Eq. (13) in the “Materials and methods” section) from the mathematical model using the multiple-scale method. The theoretical predictions of Eq. (13) are shown in Fig. 2d, e as solid green lines, which are in good agreement with the experimental results. These results suggest that the size of the peak frequency-shift of R1 from locking to unlocking can be elevated by increasing the initial frequency offset or the coupling strength between two resonators. This would contribute to a high SNR and thus high detection resolution for frequency unlocking-based bifurcation binary sensors.

## Unlocking-based bifurcation MEMS sensors

### Detection mechanisms

We propose a novel bifurcation sensor that exploits the sudden transition from locking to unlocking, resulting in a large increase in the peak frequency in coupled resonators under an IR regime. The primary principle of this sensor is to relate a detectable shift in the peak frequency of resonator R1 from locked to unlocked to a target stimulus change. Since frequency-locking arises due to IR between two coupled resonators, the onset of frequency unlocking is triggered by breaking that IR. In addition to increasing the excitation amplitude, two other approaches can be deployed to achieve this purpose. The first is to increase the frequency ratio of resonators R1 and R3 beyond 1:3, by increasing the frequency of R1 or reducing the frequency of R3. This approach suggests the possibility of detecting stimuli that affect the stiffness or mass of R1 or R3, such as changes in a potential field force or the addition of mass. The second approach is to reduce the coupling voltage to shrink the frequency locking region such that the resonator R1, initially operating at the critical point (Point A in Fig. 2c), will exit that region and unlock. This approach allows the sensor to act as an electrometer for charge detection.

**Fig. 3:**
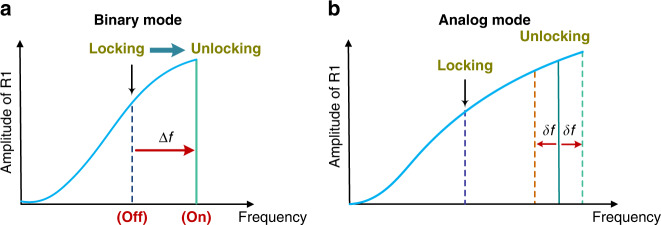
**Detection mechanism for unlocking-based bifurcation sensors.** **a** The binary mode exploits the qualitative difference between the peak frequency before and after unlocking, reporting On-state for target stimuli above the threshold and OFF-state for stimuli below the threshold. **b** The analog mode exploits the peak frequency shift after unlocking

The proposed detection mechanism allows the sensor to operate in binary or analog mode. This is achieved by observing the jump in the peak frequency and using it to detect a target stimulus in excess of a given (safe) threshold or by creating a calibration curve to relate the peak frequency to the amount of change in stimulus:

- A binary detector where the OFF (0) signal would correspond to the locked peak frequency while the ON (1) signal would correspond to a discrete and significant shift in peak frequency after unlocking, as shown in Fig. 3a. This operation mode is more robust due to the high SNR provided by the frequency jump.
- An analog sensor where a one-to-one relationship between the peak frequency after unlocking and the target stimulus magnitude is established and used as a calibration curve to quantify continuous stimulus changes, as shown in Fig. 3b. This operation mode requires a precise readout circuit for the quantification of the measurand.

### Charge detection

**Fig. 4:**
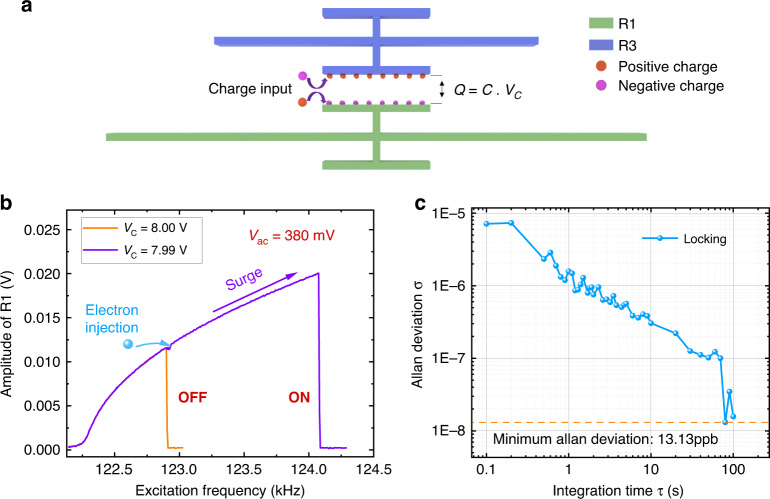
**Charge detection in binary mode.** **a** A schematic diagram of the charge detection. **b** The peak frequency before (OFF) and after (ON) the coupling voltage reduction (electron injection) of 0.01 V. **c** Allan deviation of the locked peak frequency of resonator R1

To validate the proposed sensor detection paradigm, we conducted an experimental demonstration of charge detection using the coupled MEMS resonators. The schematic of the proposed electrometer is shown in Fig. 4a. The external charge input $\Delta Q$ collected at the coupling parallel plates will cause a change in the coupling voltage $V_C$, as per $\Delta Q = C\Delta V_C$ (where $C = 0.0137707$ pF is the capacitance between two resonators’ coupling parallel plates, described in Supplementary S6). Injecting a positive charge to the negatively charged plate, and vice versa, reduces the total charge between the plates, thereby deprecating the voltage across them and, consequently, decreasing the coupling strength between the resonators. This will trigger the onset of the frequency unlocking of R1, initially operating near the critical bifurcation point. The resulting significant shift in the peak frequency from locking to unlocking can be used to create discrete output states, realizing a binary charge detector. On the other hand, the quantitative changes in peak frequency after unlocking due to coupling voltage changes can be exploited to realize an analog electrometer.

#### Binary mode

In the binary mode, the initial coupling voltage between the two resonators is set to 8 V. The locked frequency of resonator R1 is remeasured as $f_{ir} = 122.898$ kHz due to the frequency drift of R3 under different room temperatures. A manual forward sweep starting from $V_{ac} = {\kern 1pt} 360$ mV was carried out with an excitation voltage step of 1 mV to obtain a better estimate of the critical excitation amplitude $V_{crit}$ under ambient external disturbances. We note that all the excitation amplitudes within the frequency**-**locking region can be selected as the operating point, the detection resolution, however, increases as the operating amplitude of the excitation force is brought closer to the critical bifurcation point. This closeness is bounded by the uncertainty of the critical excitation amplitude, which may inadvertently activate the frequency-unlocking event, thereby causing false-positives. Therefore, the closest operating excitation amplitude of the sensor should exclude this uncertainty region to protect against false-positives. We define an operational set-off excitation amplitude as $\delta V_{ac}$ = $V_{crit} - V_{op}$, where $V_{op}$ is the operating excitation amplitude. A stability study was carried out to determine the closest operating point by increasing the set-off excitation amplitude $\delta V_{ac}$ in steps of 1 mV. A set-off excitation amplitude was declared stable if it can provide consistent response in 10 independent tests. We found the minimum realizable set-off excitation amplitude for the experimental setup to be $\delta V_{ac} = 3{\mathrm{mV}}$.

As a safety margin and to consider a higher confidence level, we set $\delta V_{ac} = 5 {\mathrm{mV}}$, yielding an operating excitation amplitude $V_{op} = 380{\kern 1pt} {\mathrm{mV}}$. The external charge input is imitated by decreasing the coupling voltage. A forward frequency sweep starting from $f = 122$ kHz is carried out to track the peak frequency until it finds a qualitative (sharp) drop in the amplitude and then recalls that frequency as the peak frequency. As the coupling voltage drops by 10 mV, from 8 V to 7.99 V, the peak frequency unlocks and undergoes a sudden surge of $\Delta f = 1177$ Hz, as shown in Fig. 4b. This indicates the detection threshold (minimum detectable charge) of the sensor at the set-off excitation amplitude of 5 mV to be $1.37 \times 10^{ - 16}{\kern 1pt} {\kern 1pt} {\kern 1pt} C$, as per $\Delta Q = C\Delta V_C$. Any charge change in excess of this threshold is detected.

The uncertainty of the critical excitation amplitude arises from the fluctuations of the excitation voltage, the locked frequency and the natural frequency of R1. The latter is dominant and is caused by the additional effect of Joule heating introduced to tune the natural frequency of R1, such that $3\omega _1 < \omega _3$, in our experiment. It could be mitigated by introducing eligible resonator designs. In that case, the minimum detectable charge would be mainly determined by the locked frequency stability. We evaluate the frequency stability in the locking region via Allan deviation in Fig. 4c. To this end, we use a LIA-based phase locked loop (PLL) to track a time series of the locked frequency using a fixed sampling rate of 7Sa/sec for a duration of 300 s. The minimum Allan deviation of the locking frequency is observed to be 13.13 ppb, yielding extremely small frequency fluctuations of 0.0016 Hz. This indicates that the proposed binary electrometer would achieve an extraordinarily high resolution, provided that the heating induced natural frequency fluctuation in the frequency sweep process can be eliminated.

#### Analog mode

**Fig. 5:**
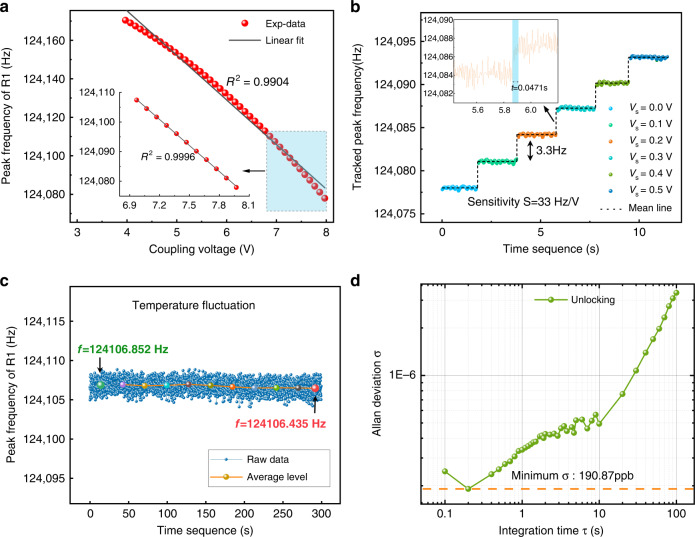
**Charge detection in analog mode.** **a** Measured peak frequencies of R1 after unlocking vs. the coupling voltage. **b** The real-time detection ladder diagram as the coupling voltage varies in steps of 0.1 V. The markers represent the tracked real-time peak frequency by exploiting a PLL, and the solid line denotes the mean value in one test. Inset: zoom on a detection ladder. **c** The time series of a specific peak frequency over 5 minutes. The colored markers represent the average frequency over 200 adjacent frequency samples. **d** Allan deviation of the peak frequency of R1 after unlocking

In the analog mode, a closed-loop circuit-based PLL is set to track the peak frequency for real-time charge quantitative detection. Details of the closed-loop procedures are described in Supplementary S7. The peak frequencies of R1 after unlocking are shown in Fig. 5a as the coupling voltage is varied in steps of 0.1 V. The peak frequency increases linearly as the coupling voltage decreases with a fitting coefficient of $R^2$ = 0.9904 and a dynamic range of 4 V, which can serve as the calibration curves to quantitatively detect charge changes. Figure 5(b) shows the real-time detection ladder diagram as the coupling voltage varies in steps of 0.1 V, which is equivalent to the charge variation of $1.37 \times 10^{ - 15}C$. The dotted black line corresponds to the average peak frequency. The detection time for a voltage step of 0.1 V is only 0.047 s, as shown in the inset of Fig. 5b. The real-time detection ladder diagram with steps of 0.01 V is also shown in Supplementary S8. The sensitivity $S$ (scaling factor), defined as the ratio of the shift in peak frequency to the change in coupling voltage, was found to be 33 Hz/V.

To examine the impact of temperature drift on the stability of the peak frequency in a closed-loop circuit, we measured the time series of a specific peak frequency over 5 minutes, Fig. 5c. The colored markers are the average frequencies over 200 adjacent points. A frequency fluctuation of 0.4 Hz is observed within 5 minutes, which indicates that the temperature effect is negligible in our real-time detection.

The limit of detection (LOD) or charge resolution $R$ can be obtained as the ratio of the minimum detectable frequency shift $\delta f$ to the sensitivity $S$, such as $R = C \cdot \delta f/S$. The minimum detectable frequency change is dependent on the frequency fluctuation and can be determined by evaluating the Allan deviation $\sigma _A(\tau )$ of the characteristic frequency $f_p$ as $\delta f = f_p \cdot \sigma _A(\tau )$. Figure 5(d) shows the Allan deviation $\sigma _A$ of the tracked peak frequency of R1 as a function of the integration time $\tau$. The calculated minimum Allan deviation is 190.83 ppb, yielding a charge resolution of $101.48 \times 10^{ - 19}C$ equivalent to 63 electrons.

Table S1 in Supplementary S9 lists comparisons of the state-of-the-art resonant electrometers^33,39,40^ in terms of the charge resolution, response time, and power consumption. In comparison, the electrometer operating in analog mode from this work achieves a moderate charge resolution and relatively shorter response time. However, the potential of our detection mechanism is not fully realized due to the large size of the resonators^4^ and the limitations of the real-time frequency tracking PLL circuit^41^ and measurement circuit we used in the experiment. If a smaller-scale resonator was used under an experimental condition with a higher-end PLL and measurement circuits, the proposed detection mechanism would have a better charge resolution in the analog mode.

## Conclusions

We report a new bifurcation-based sensor paradigm exploiting the frequency unlocking associated with IR between two electrostatically coupled micro-resonators. This sensor can function as a binary detector of a target stimulus in excess of a given (safe) value by monitoring the jump in peak frequency from locking to unlocking. It can also serve as an analog sensor to quantify the stimulus change explicitly by measuring the continuous change in peak frequency beyond unlocking. The proposed sensor enables the detection of stimuli that affect the stiffness or mass of the resonators, such as changes in a potential field force or the addition of a mass. It can also act as an electrometer for charge sensing.

The frequency locking and unlocking behaviors under IR regime were investigated experimentally. A closed-form formula of the size of the peak frequency shift from locking to unlocking, upon crossing the bifurcation point, was derived and validated experimentally. We found that a larger frequency-shift from locking to unlocking and, therefore, a higher SNR and resolution for the binary detector can be attained by designing resonators with a larger initial frequency offset or by increasing the coupling strength between them.

We demonstrated the feasibility of the proposed detection mechanism for charge detection. Sweep-based peak tracking was utilized to estimate the frequency-jump event in binary mode, while a closed-loop circuit was built for real-time charge detection in analog mode. The realized minimum detectable charge in binary mode is $1.37 \times 10^{ - 16}C$ and in analog mode is $1 \times 10^{ - 17}C$. While the binary sensor had no upper limit for its dynamic range, the upper limit of the analog sensor was $5.48 \times 10^{ - 14}C$.

Compared to traditional bifurcation sensing mechanisms, the proposed mechanism has the advantages of (i) a remarkable SNR in binary mode because of the large jump across the bifurcation in peak frequency, (ii) excellent frequency stability in both binary and analog modes provided by IR, and (iii) the flexibility to operate the sensor in either binary or analog mode. The detection resolution in binary mode is dependent on the minimum realizable set-off excitation amplitude, which is affected by the uncertainty of the excitation voltage and R1’s natural frequency. In our experiment, Joule heating, which is introduced to tune the natural frequency of R1 for IR, was the main driver of this uncertainty. The detection resolution in binary mode could be significantly improved by designing the resonators to satisfy the condition $3\omega _1 < \omega _3$ or by an active temperature compensation circuit. We plan to investigate those approaches in future work.

## Materials and methods

### Theoretical analysis

We adopt a lumped-mass model that describes the transverse displacements of two electrostatically coupled micro-resonators by the governing nondimensional equations of motion^42,43^:

1 $$\begin{array}{l}\ddot x_1 + x_1 = - 2\gamma _1\dot x_1 - \beta _1x_1^3 - 3g_1x_1^2x_3 + f_1\cos (\Omega t)\\ \ddot x_3 + \omega _3^2x_3 = - 2\gamma _3\dot x_3 - g_3x_1^3\end{array}$$

where $x_i$, $\omega _i$ and $\gamma _i(i = 1,3)$ are the transverse displacements, natural frequency, and damping ratio of resonators R1 and R3, respectively. $\beta _1$ and $f_1$ are the cubic stiffness and driving amplitude of R1, respectively, while $g_1$ and $g_3$ represent the coupling coefficients which are proportional to the square of the coupling voltage $V_C$. The derivation of Eq. (1) is presented in Supplementary S5.

We use the perturbation method of multiple scales^27^ to solve Eq. (1). The general approximate solution of Eq. (1) is given by:

2 $$\begin{array}{l}x_1(\tau _0,\tau _1) = A_1(\tau _1)\cos [\omega _1\tau _0 + \theta _1(\tau _1)],\\ x_3(\tau _0,\tau _1) = A_3(\tau _1)\cos [\omega _3\tau _0 + \theta _3(\tau _1)],\end{array}$$

where $\tau _n = \varepsilon ^nt$. We define $\tau _0 = t$ as the fast variable while $\tau _1 = \varepsilon t$ as the slow variable. $A_i$ and $\theta _i{\kern 1pt} {\kern 1pt} {\kern 1pt} (i = 1,{\kern 1pt} {\kern 1pt} {\kern 1pt} 3)$ are the motional amplitude and phase difference of R1 and R3, respectively. Considering the case of primary resonance, we define the excitation frequency $\Omega$ and the natural frequency $\omega _3$ of R3 in terms of the small detuning parameters $\delta _1$ and $\delta _3$, respectively, with respect to the natural frequency as:

3 $$\begin{array}{l}\Omega = 1 + \varepsilon \delta _1\\ \omega _3 = 3 + \varepsilon \delta _3\end{array}$$

Next, we expand the equations of motion, Eq. (1), in powers of $\varepsilon$. Then, the equations describing the amplitude and phase modulations are obtained by eliminating the secular terms. The first-order approximation of the solutions yields^45^

4 $$\begin{array}{l}A_1^\prime = - r_1A_1 - \frac{{3g_1A_1^2A_3\sin \phi _3}}{8} + \frac{{f_1\sin \phi _1}}{2}\\ A_1\theta _1^\prime = \frac{{3g_1A_1^2A_3\cos \phi _3}}{8} + \frac{{3\beta _1A_1^3}}{8} + \frac{{f_1\cos \phi _1}}{2}\\ \omega _3A_3^\prime = - r_3\omega _3A_3 + \frac{{g_3A_1^3\sin \phi _3}}{8}\\ \omega _3A_3\theta _3^\prime = \frac{{g_3A_1^3\cos \phi _3}}{8}\end{array}$$

where the prime symbol denotes the derivative with respect to the slow time $\tau _1$, and the phase angles $\phi _1$ and $\phi _2$ are defined as

$$\phi _1 = \delta _1\tau _1 - \theta _1,\;\phi _3 = \delta _3\tau _1 + \theta _3 - 3\theta _1$$

To obtain the steady-state response, we set the slow time derivatives to vanish. Setting $\phi _1^\prime {{{\mathrm{ = }}}}0$, $\phi _3^\prime {{{\mathrm{ = }}}}0$ yields $\theta _1^\prime = \delta _1$, $\theta _3^\prime = 3\delta _1 - \delta _3$, while setting $A_1^\prime = A_3^\prime = 0$ gives the following algebraic equations for the stationary solutions:

5 $$\begin{array}{l}8r_1A_1 + 3g_1A_1^2A_3\sin \phi _3 - 4f_1\sin \phi _1 = 0\\ 8\omega _1A_1(\Omega - 1) - 3g_1A_1^2A_3\cos \phi _3 - 3\beta _1A_1^3 + 4f_1\cos \phi _1 = 0\\ 8r_3\omega _3A_3 - g_3A_1^3\sin \phi _3 = 0\\ 8\omega _3A_3(3\Omega - \omega _3) - g_3A_1^3\cos \phi _3 = 0\end{array}$$

Substituting the last two equations of Eq. (5) into its first two equations yields the algebraic equations describing the stationary response of R1, only with respect to $A_1$ and $\phi _1$

6 $$\begin{array}{l}8r_1A_1 + \frac{{3r_3\omega _3g_1g_3A_1^5}}{{8\omega _3^2[(3\Omega - \omega _3)^2 + r_3^2]}} - 4f_1\sin \phi _1 = 0\\ 8A_1(\Omega - 1) - \frac{{3g_1g_3(3\Omega - \omega _3)A_1^5}}{{8\omega _3^2[(3\Omega - \omega _3)^2 + r_3^2]}} - 3\beta _1A_1^3 + 4f_1\cos \phi _1 = 0\end{array}$$

IR occurs when $3\Omega \approx \omega _3$, where a frequency gap occurs because Eq. (6) does not provide a (real) solution for $\phi _1$^44^. This can be seen in:

7 $$\left| {\sin \phi _1} \right| = \left| {(8r_1A_1 + \frac{{3r_1\omega _3g_1g_3A_1^5}}{{8\omega _3^2[(3\Omega - \omega _3)^2 + r_3^2]}})\frac{1}{{4f_1}}} \right| > 1$$

As the excitation amplitude $f_1$ is further increased, the right-hand side of Eq. (7) decreases gradually until its solution becomes real. The critical excitation amplitude$f_c$ required to eliminate this frequency gap and unlock the frequency can be calculated by setting $\sin \phi _1 = 1$:

8 $$f_c = \frac{1}{4}(8r_1A_1 + \frac{{3g_1g_3A_1^5}}{{8\omega _3r_3}}) = 2r_1A_1 + \frac{{3g_1g_3A_1^5}}{{32\omega _3r_3}}$$

Under this critical excitation amplitude, the nonlinear resonance (peak) frequency $\Omega _p$ of R1 after unlocking can be evaluated using the amplitude-frequency relation in the Duffing oscillator equation^13^ as:

9 $$\Omega _p{{{\mathrm{ = }}}}\frac{{3\beta _1}}{8}A_p^2 + 1 = \frac{{3\beta _1}}{8}\left(\frac{{f_c}}{{2\gamma _1}}\right)^2 + 1$$

where $A_p$ is the amplitude of R1 after unlocking. Substituting Eq. (8) into Eq. (9) and retaining terms up to the sixth-order in $A_1$ yields

10 $$\Omega _p = \frac{{3\beta _1}}{8}(A_1^2 + \frac{{3g_1g_3A_1^6}}{{32r_1r_3\omega _3}}) + \omega _1$$

where the amplitude $A_1$ of resonator R1 can be assumed as

11 $$A_1 = \sqrt {\frac{8}{{3\beta _1}}\sigma }$$

where $\sigma = \frac{{\omega _3}}{3} - 1$ is the initial frequency offset of R1 between its natural frequency and the locked frequency. Substituting Eq. (11) into Eq. (10) yields

12 $$\Omega _p = \sigma + \frac{{2g_1g_3\sigma ^3}}{{3\gamma _1\gamma _3\omega _3\beta _1^2}} + 1$$

Then, the frequency shift $\Delta \omega$ from locking to unlocking is given by:

13 $$\Delta \omega = \Omega _p - \frac{{\omega _3}}{3} = \frac{{2g_1g_3\sigma ^3}}{{9\gamma _1\gamma _3(1 + \sigma )\beta _1^2}}$$

Equation (13) captures the dependence of the peak frequency-jump on the initial frequency offset, coupling strength and material properties.

## Supplementary information

Supplemental Material
