# Supplementary material for: Frequency unlocking-based MEMS bifurcation sensors
Source: Microsyst Nanoeng. 2023 May 16;9:58. doi: 10.1038/s41378-023-00522-2 (PMC10185684; doi:10.1038/s41378-023-00522-2)
Supplement: Supplementary file 1 — Supplemental Material [file 41378_2023_522_MOESM1_ESM.docx]

Supplementary Materials for

**Frequency unlocking-based MEMS bifurcation sensors**

Yan Qiao^1,a^, Zhan Shi^2,a^, Yutao Xu^3^, Xueyong Wei^3^, Alaaeldin Elhady^4^, Eihab Abdel-Rahman^4^, Ronghua Huan^2, *^, Wenming Zhang^1,*^

*^1^State Key Laboratory of Mechanical System and Vibration, School of Mechanical Engineering, Shanghai Jiao Tong University, Shanghai, China*

*^2^Department of Mechanics, Key Laboratory of Soft Machines and Smart Devices of Zhejiang Province, Zhejiang University, Hangzhou, China*

*^3^State Key Laboratory for Manufacturing Systems Engineering, Xi’an Jiaotong University, Xi’an, China*

*^4^Department of Systems Design Engineering, University of Waterloo, Waterloo, ON, Canada*

^*^ *Correspondence*: [wenmingz@sjtu.edu.cn](mailto:wenmingz@sjtu.edu.cn) (W.Zhang) rhhuan@zju.edu.cn (R.Huan)

**Contents**

**S1. Device Fabrication Process**

**S2. Structure of the Resonators**

**S3. Experimental Setup**

**S4. Schematic of the Piezoresistive Detection**

**S5. Theoretical Analysis**

**S6. The Influence of Fringe Effect**

**S7. Peak Frequency Tracking Using a Phase Locked Loop (PLL)**

**S8. Real-time Charge Detection**

**S9. Comparisons of the Electrometers Performance**

**S1. Device Fabrication Process**


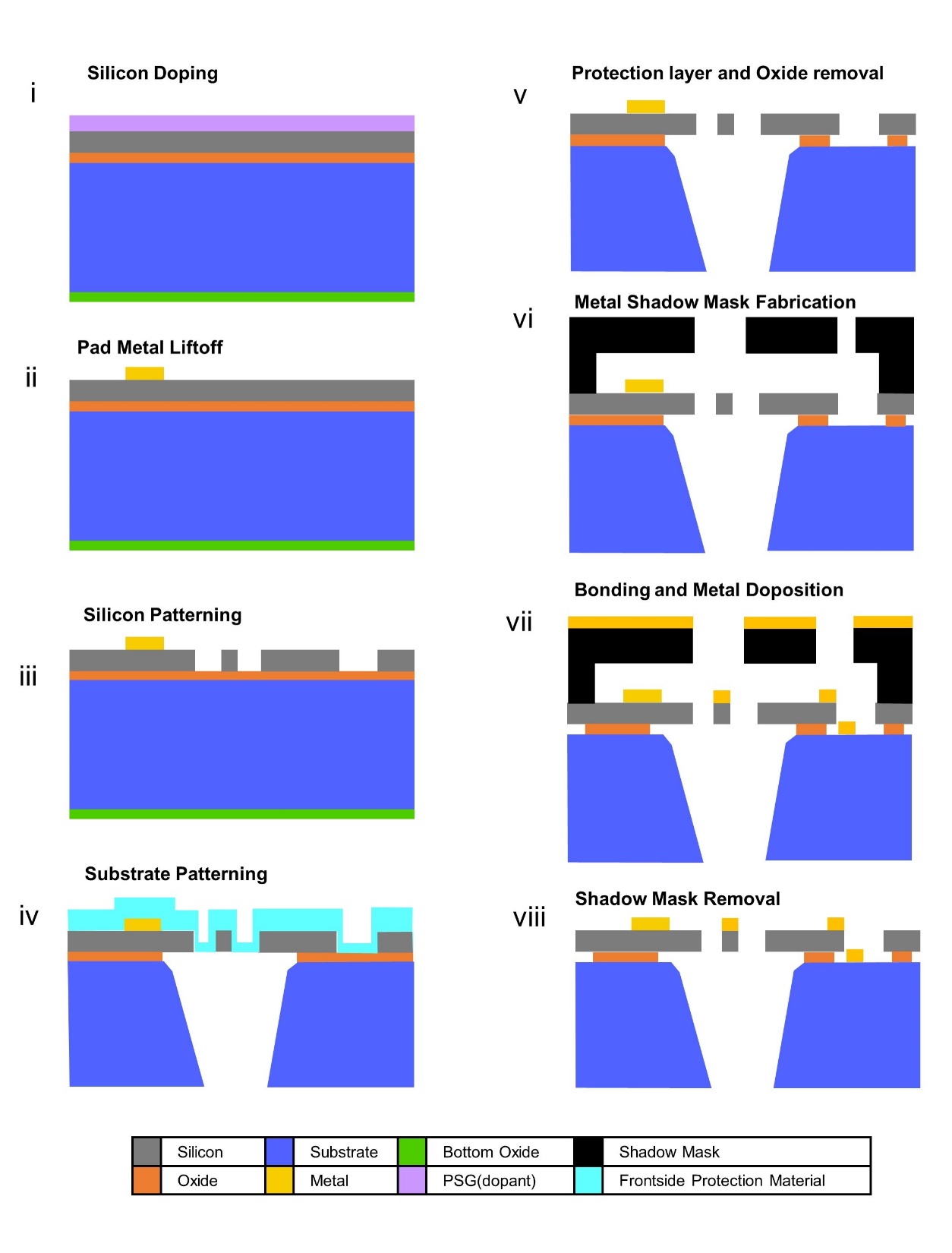


Fig.S1 SOI-MUMPs micromachining process.

The micro-resonators are fabricated through a commercial silicon-on-insulator (SOI)–MUMPs micromachining process^1^. A 25um SOI wafer is used as the starting substrate. The fabrication process lies in 8 steps:

(i) A phosphosilicate glass layer (PSG) is deposited, then drive the Phosphorous dopant into the top surface of the Silicon layer. The PSG layer is subsequently removed using wet chemical etching.

(ii) Exposing the photoresist with light through the first level mask (PADMETAL), and then developing it. A metal stack is deposited over the photoresist pattern by e-beam evaporation in the opened areas.

(iii) Exposing the photoresist to UV light through the second level mask (SOI), and then developing it. Deep reactive ion etching (DRIE) is used to etch the Silicon down to the Oxide layer.

(iv) Reactive ion etching (RIE) is used to remove the Bottom Side Oxide layer. A wet oxide etch process is then used to remove the Oxide layer in the regions defined by the TRENCH mask.

(v) The frontside protection material is then stripped using a dry etch process. The remaining “exposed” Oxide layer is removed from the top surface using a vapor HF process.

(vi) A separate silicon wafer is used to fabricate a shadow mask for the Metal pattern. DRIE silicon etching is used to etch completely through the shadow mask wafer.

(vii) The shadow mask is aligned and temporarily bonded to the SOI wafer.

(viii) The shadow mask is removed, leaving a patterned Metal layer on the SOI wafer.

**S2. Structure of the Resonators**

Fig.S2 shows the microscopic image of the electrostatically coupled micro-resonators. The two micro-resonators R1 and R3 are designed as two double-ended tuning forks (DETFs) due to their significant advantages, such as energy dissipation minimization and common-mode rejection of acceleration. The endings of the tuning fork are anchored in gold electrodes to connect with external electrical sources. The gold electrodes in the mid-span are utilized for electrostatic actuation. The plates between two tuning forks are used to produce electrostatic coupling. A schematic of the micro-resonators is shown in Fig. S3. A summary of the dimensions and design parameters assigned for the resonators is given in Table S1.


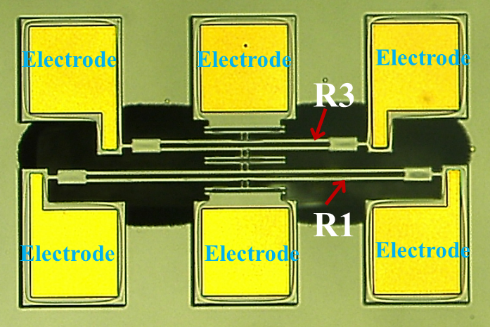


Fig. S2 Microscopic image of the coupled micro-resonators.


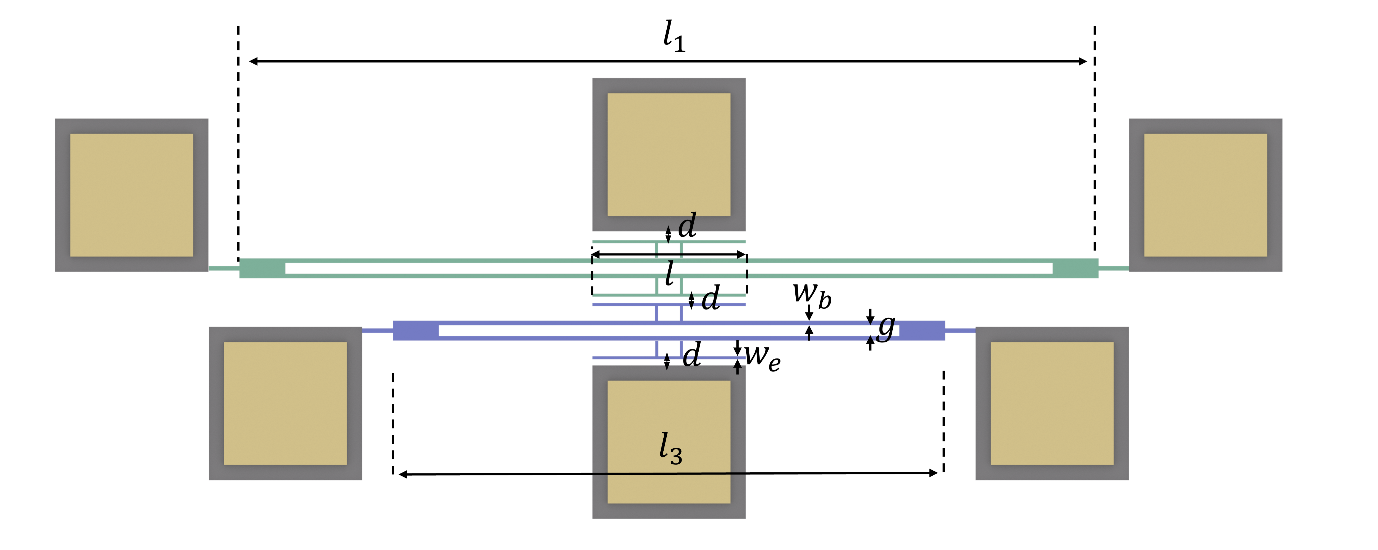


Fig.S3. Schematic view of the coupled micro-resonators.

| **Table S1.** Dimensions of the electrostatically coupled micro-resonators | |
| --- | --- |
| **Parameters** | **Dimensions /** $\mu m$ |
| Device thickness ***h*** | 25 |
| R1 length $l_{1}$ | 550 |
| R3 length $l_{3}$ | 292 |
| Beam width ***w_b_*** | 7 |
| Electrode length ***l*** | 150 |
| Electrode width ***w_e_*** | 5 |
| Gap between beams ***g*** | 6 |
| Gap between coupling electrodes ***d*** | 3 |
| Gap between resonator and the driving electrode ***d*** | 3 |

**S3. Experimental Setup**


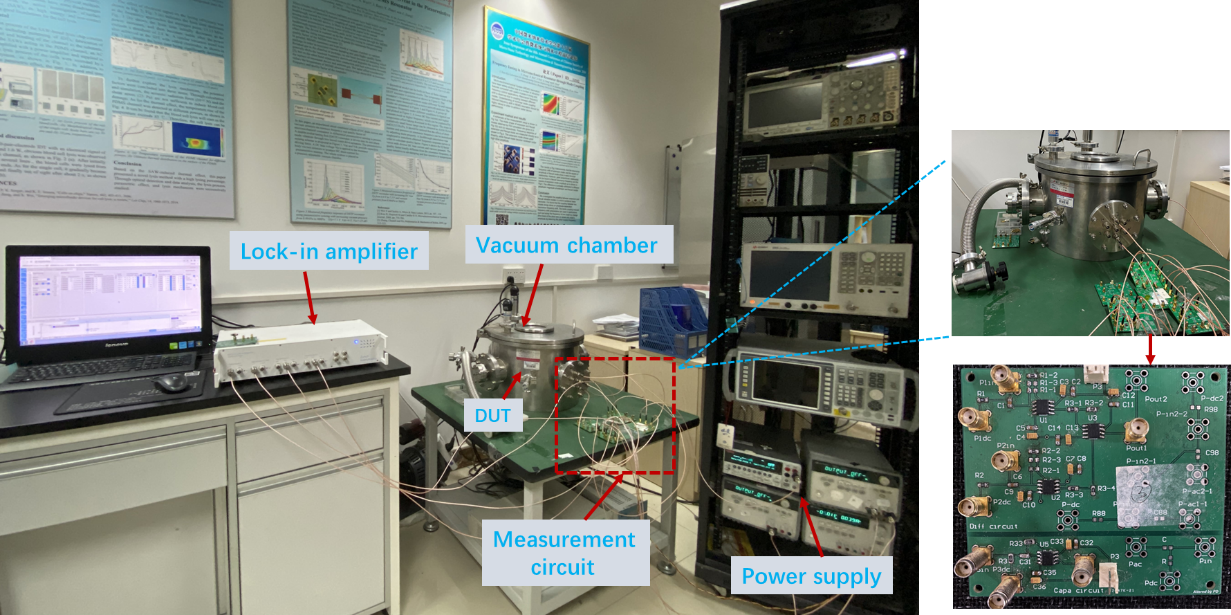


Fig. S4 Experimental setup.

**S4. Schematic of the Piezoresistive Detection**


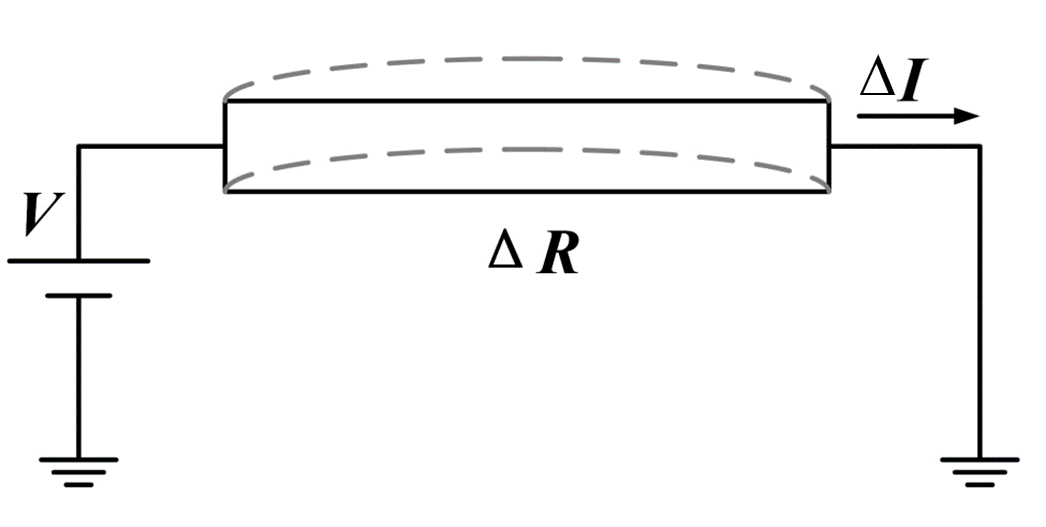


Fig.S5 Schematic of the piezoresistive detection mechanism.

The electric potential difference between the two ends of the resonator remains constant, while the vibration of the resonator to electrostatic driven force will change resonator’s resistance ($\Delta R$) due to the piezoresistive effect, thereby causing the changes of the current$\Delta I$passing through it. Therefore, the vibration amplitude of the resonator can be measured through monitoring the dynamic current.

**S5. Theoretical Analysis**

The basic characteristics of the electrostatically coupled resonators shown in Fig. 1, indicate that the low frequency resonator (R1) exhibits Duffing-like nonlinearity while the high frequency resonator (R3) is essentially linear within their respective motion domains. A generic model capable of describing the dynamics of this system can be formulated as^2,3^

where $F_{1}$ and $F_{c}$ are the external electrostatic excitation force and the electrostatic coupling force generated by the parallel plates, respectively, which can be expressed as

Where $\tilde{x}$ and $\tilde{y}$ are the in-plane displacements of R1 and R3, respectively. $M_{1,3}$, $C_{1,3}$ and $K_{1,3}$ are the mass, damping coefficient and linear stiffness coefficient of two resonators, respectively. $K_{n}$ is the Duffing nonlinear stiffness coefficient of R1. *S* is the area of driving or coupling electrodes and $\epsilon_{r}$ is the dielectric constant. The capacitive gaps between the resonator and the drive electrode, or between the two coupling electrodes are *d*. $V_{C}$ is the coupling voltage between two resonators. The resonator R1 is excited via a harmonic voltage signal where $V_{DC}$, $V_{AC}$ and $\omega$ are the bias DC voltage, AC voltage amplitude and angular frequency, respectively. Assuming small amplitude AC drive ( $V_{DC}\gg V_{AC}$), utilizing Taylor series expansion and ignoring trivial terms, Eqs. (s2) and (s3) are simplified as

Introducing the following nondimensional transformations:

$$\begin{aligned} \sqrt{\frac{K_{1}}{M_{1}}}=\omega_{1}, \tau=\omega_{1}t,\sqrt{\frac{K_{3}}{M_{3}}}=\omega_{3},x=\frac{\tilde{x}}{d},y=\frac{\tilde{y}}{d},\gamma_{1}=\frac{C_{1}}{{2M}_{1}\omega_{1}}, \\ n=\frac{\omega_{3}}{\omega_{1}},\gamma_{3}=\frac{C_{3}}{2M_{3}\omega_{3}}, \beta_{1}=\frac{K_{n}d^{2}}{M_{1}\omega_{1}^{2}},g_{1}=\frac{S\epsilon_{r}V_{C}^{2}}{M_{1}\omega_{1}^{2}d^{3}}, \\ g_{3}=\frac{S\epsilon_{r}V_{C}^{2}}{M_{3}\omega_{1}^{2}d^{3}},\Omega=\frac{\omega}{\omega_{1}},f_{1}=\frac{S\epsilon_{r}V_{AC}V_{DC}}{M_{1}\omega_{1}^{2}d^{3}},\frac{g_{1}}{g_{3}}=\frac{M_{3}}{M_{1}} \end{aligned}$$

where $n$ is the natural frequency ratio of R1 and R3. Substituting with them into Eq. (s1), we obtain the nondimensional equation of motion as

We consider the effective terms of the expansion of the coupling terms $g_{i}(x-y)$ and $g_{i}(x-y)^{3}$ (i=1,3). Most of them are non-resonant and will not survive the averaging process, since they are composed of fast oscillating terms after the transformation^3^. For those items retained in the average process, those low-order coupling potential terms of the form $x^{n}y^{m}$,$m+n<4$ do not promote energy exchange between two resonators via amplitude modulations, and they only shift the frequencies of the corresponding resonator in parameter space^4^. When $m+n=4$, there are three coupling potential terms can be survived $H_{cp}=gx^{3}y+gx^{2}y^{2}+gxy^{3}$. However, these terms $gx^{2}y^{2}, gxy^{3}$are typically dispersive, i.e., introducing additional energy-dependent frequency-shifts to each resonator but no energy transformation and resonant interactions^5^. Only the term $gx^{3}y$can provide the energy transfer between two resonators. The corresponding coupling force is $3gx^{2}y$for resonator R1 and $gx^{3}$ for resonator R3. Then the equation of motion, Eq.(s6) can be simplified as the normal form

**S6. The Influence of Fringe Effect**

An ideal parallel plate capacitance can be expressed by

where $\epsilon_{r}$ is the permittivity.

Consider the fringe effect, a modified capacitance-computing equation is described as^6^:

With the relative parameters as shown in Table S1, the ideal capacitance $C_{0}$ is calculated as 0.01106 pF while the modified capacitance is 0.0137pF. In our paper, we adopt the latter to get a more precise result.

**S7. Peak Frequency Tracking Using a Phase Locked Loop (PLL)**

In order to track the peak frequency in analog mode in a closed-loop configuration, we performed the following steps: i) A forward frequency sweep was carried out to excite the resonator at a frequency slightly ahead of the setpoint peak frequency. ii) A built-in phase locked loop (PLL) of the Lock-in Amplifier (HF2LI -Zurich Instruments) was utilized to lock the resonator at its phase (90°) and, therefore establish a stable self- sustained oscillation. iii) The phase delay of PLL was manually adjusted with an increasing step of 0.1° to track the frequency until it reaches the maximum indicating the resonator is navigated to the peak, where the maximum frequency is the detected peak frequency. Continuous and repeated charge detection then can be carried out.

**S8 Real-time Charge Detection**

Fig.S6 **The real-time detection ladder diagram** as the coupling voltage varies in steps of 0.01 V (equivalent the charge variation of 0.137 fC). The markers represent the tracked real-time peak frequency by exploiting a PLL, and the solid line denotes the mean value in one test. Inset: the response time for a charge variation of 0.137 fC.

**S9. Comparison Chart of the Electrometers Performance**

Table 1: Comparisons of the state-of-the-art electrometers

| Year[Ref.] | Size(length×width×height) | Response time(ms) | Power(w) | Resolution(e/√Hz) | Detection mode |
| --- | --- | --- | --- | --- | --- |
| 2017^7^ | 1500×1000×25$\mu m^{3}$ | - | - | 203750 | Analog |
| 2018^8^ | 1425×1050×25$\mu m^{3}$ | - | - | 16250 | Analog |
| 2018^9^ | 1280×2700×30$\mu m^{3}$ | 150 | - | 9.21 | Analog |
| 2020^10^ | 350×175×25$\mu m^{3}$ | - | - | 0.197 | Analog |
| 2020^11^ | 127.3×127.3×25$\mu m^{3}$ | 20.9 | - | 0.17 | Analog |
| 2022^12^ | 127.3×127.3×25$\mu m^{3}$ | - | - | 68 | Analog |
| **This work** | **550×320×25**$\mu m^{3}$ | **47.1** | **0.32** | **A: 63** | **Analog, Binary** |

Note: The **Size** in this table represents the dimension of the vibrational part of the device. In literature ^8, 9^, and ^10^, the specially designed actuating and sensing electrodes are included.

**Reference**

1. Cowen A, et al. SOIMUMPs design handbook. MEMSCAP Inc. 2011.
2. Wang, X. et al. Amplitude region for triggering frequency locking in internal resonance response of two nonlinearly coupled micro-resonators. *Int. J. Nonlin. Mech.* **130**, doi:10.1016/j.ijnonlinmec.2021.103673 (2021).
3. Wang, X. *et al.* Frequency locking in the internal resonance of two electrostatically coupled micro-resonators with frequency ratio 1: 3. *Mech. Syst. Signal Pr.* **146**, 106981 (2021).
4. Shoshani, O. & Shaw, S. W. Resonant modal interactions in micro/nano-mechanical structures. *Nonlinear Dyn.* **104**, 1801-1828 (2021).
5. Güttinger, J. *et al.* Energy-dependent path of dissipation in nanomechanical resonators. *Nat. Nanotechnol*. **12**, 631-636 (2017).
6. Dong, L., Li, Y., Yan, H. & Sun, L. Effects of Electric Field Fringe on Performances of Grid Strip Capacitive MEMS Devices. *High Technology Letters*.
7. Chen, D., Zhao, J., Wang, Y. & Xie, J. An electrostatic charge sensor based on micro resonator with sensing scheme of effective stiffness perturbation. *J. Micromech. Microeng.* **27**, 065002 (2017).
8. Chen, D., Zhao, J., Wang, Y., Xu, Z. & Xie, J. Sensitivity manipulation on micro-machined resonant electrometer toward high resolution and large dynamic range. *Appl. Phys. Lett.* **112**, 013502, (2018).
9. Yang, J., Kang, H. & Chang, H. A micro resonant electrometer with 9-electron charge resolution in room temperature. *2018 IEEE Micro Electro Mechanical Systems (MEMS)*. 67-70 (2018).
10. Wang, X., Wei, X., Pu, D. & Huan, R. Single-electron detection utilizing coupled nonlinear microresonators. *Microsyst. Nanoeng.* **6**, 78, (2020).
11. Chen, D. et al. Ultrasensitive Resonant Electrometry Utilizing Micromechanical Oscillators. *Phys. Rev. Appl.* **14**, 014001, (2020).
12. Chen, H., Chen, D., Shi, Z., Huan, R. & Xie, J. A MEMS frequency modulation electrometer based on pre-bias charge mechanism to enhance performance. *J. Micromech. Microeng.* **32**, 105003, (2022).
